# Supramolecular Gels Incorporating *Cordyline terminalis* Leaf Extract as a Polyphenol Release Scaffold for Biomedical Applications

**DOI:** 10.3390/ijms22168759

**Published:** 2021-08-16

**Authors:** Dieu Phuong Nguyen Thi, Dieu Linh Tran, Phuong Le Thi, Ki Dong Park, Thai Thanh Hoang Thi

**Affiliations:** 1 Biomaterials and Nanotechnology Research Group, Faculty of Applied Sciences, Ton Duc Thang University, Ho Chi Minh City 700000, Vietnam; ntdp.1403@gmail.com; 2 Department of Molecular Science and Technology, Ajou University, Suwon 16499, Korea; linhtran92@ajou.ac.kr (D.L.T.); phuong0612@ajou.ac.kr (P.L.T.); kdp@ajou.ac.kr (K.D.P.)

**Keywords:** supramolecular gels, *Cordyline terminalis*, polyphenols, antioxidant

## Abstract

*Cordyline terminalis* leaf extract (aqCT) possesses abundant polyphenols and other bioactive compounds, which are encapsulated in gelatin–polyethylene glycol–tyramine (GPT)/alpha-cyclodextrin (α-CD) gels to form the additional functional materials for biomedical applications. In this study, the gel compositions are optimized, and the GPT/α-CD ratios equal to or less than one half for solidification are found. The gelation time varies from 40.7 min to 5.0 h depending on the increase in GPT/α-CD ratios and aqCT amount. The aqCT extract disturbs the hydrogen bonding and host–guest inclusion of GPT/α-CD gel networks, postponing the gelation. Scanning electron microscope observation shows that all gels with or without aqCT possess a microarchitecture and porosity. GPT/α-CD/aqCT gels could release polyphenols from 110 to 350 nmol/mL at the first hour and sustainably from 5.5 to 20.2 nmol/mL for the following hours, which is controlled by feeding the aqCT amount and gel properties. GPT/α-CD/aqCT gels achieved significant antioxidant activity through a 100% scavenging DPPH radical. In addition, all gels are non-cytotoxic with a cell viability more than 85%. Especially, the GPT3.75α-CD10.5aqCT gels with aqCT amount of 3.1–12.5 mg/mL immensely enhanced the cell proliferation of GPT3.75α-CD10.5 gel without extract. These results suggest that the inherent bioactivities of aqCT endowed the resulting GPT/α-CD/aqCT gels with effective antioxidant and high biocompatibility, and natural polyphenols sustainably release a unique platform for a drug delivery system or other biomedical applications.

## 1. Introduction

Hydrogels are a promising candidate for biomedical applications, especially for drug/cell encapsulation applied in regenerative medicine and tissue engineering. Hydrogels possess a three-dimensional structure, high porosity, high water content, biocompatible properties, and biodegradable ability [1,2,3]. Chemical reactions or physicochemical association-driven processes can be applied to form hydrogels. In the case of chemically crosslinking hydrogels, there has been Michael addition and click reactions, redox- or photo-polymerization, and enzymatic reactions using horseradish peroxidases (HRP), phosphatases (PP), and transglutaminases (TG) [3,4,5]. However, these chemical reactions utilized catalysts, initiators, or oxidizing agents that might interact with drugs/cells encapsulated inside hydrogels. Among them, Horseradish peroxidase-catalyzed hydrogels were formed in the mildest conditions and showed tunable mechanical properties and biocompatibility [6]. These hydrogels were applied successfully to incorporate various cell lines and sustainably release many types of drugs [3,7,8]. However, this reaction used hydrogen peroxide as a strongly oxidizing agent, which easily interacts with bioactive compounds, especially polyphenols, and thus leads to inactivate them. In the case of physically crosslinking hydrogels, the driven forces can be π-π stacking, hydrophobic interactions, *Van der Waals* interactions, hydrogen bonds, and inclusion complex formation of the biocompatible modified polymers [2]. Therefore, these conditions were favored to encapsulate bioactive compounds, cells, or other pharmaceutical products more than the chemical gels [2].

*Cordyline terminalis* L. Kunth belongs to the Asparagaceae family and is one of the tropical plants grown popularly in Asia, Australia, and the Pacific Islands [9]. It is well-known as a remedy for Asians in the treatment of various diseases, such as cough, dysentery, fever, kidney problems, Tibiae, inflammation in the digestive tract, and headache [9]. Until now, there has been little research studying this plant. M. Amzad et al. quantitatively determined the total phenolic and flavonoid contents in *Cordyline terminalis* L. Kunth leaves [9], but further studies for specific applications have yet to be carried out. Meanwhile, the bioactive compounds have been considered as one of the critical factors to synthesize or regulate the critical metabolites to prevent degenerative diseases [10]. The alternative of classic drugs with natural substances also contributes to reducing dysbiosis. A well-balanced microbiota is essential to maintain the general process of health, minimizing the occurrence of oxidative stress and neurodegeneration [10]. However, the bioactive compounds have problems during an applying procedure, such as spreading diffusion, instability, and frequently repeated doses [11]. These difficulties require an encapsulation strategy for bioactive compounds in each specific application to minimize the compound lost and thus maximizing the desired effects [11,12]. Individual natural substance is studied to understand its particular effect, but the applied research likely favors the mixture components due to the additive/synergistic effects and cost-effectiveness [13]. They could exhibit the excellent biological activities in reducing the risk of several diseases, including cancer, heart disease, stroke, Alzheimer’s, diabetes, and age-related functional decadence [11], even as a ray of hope in COVID-19 management [14]. Motivated from these reports, the crude extract of *Cordyline terminalis* Kunth leaves was chosen instead of isolated compounds.

In this study, we structured the non-covalently crosslinking gels from gelatin–polyethylene glycol–tyramine (GPT) and alpha-cyclodextrin (α-CD) for encapsulation of an aqueous *Cordyline terminalis* L. Kunth leaf extract (aqCT) to release polyphenols and investigate their antioxidant and cell biocompatible capacity. Firstly, the GPT precursor polymers were prepared with suitable PEG substitution, and the aqCT was primarily tested on the phytochemical compositions. The optimal GPT/α-CD ratios were investigated to form the supramolecular gels and incorporate the various amounts of aqCT. The intra-morphology of gels was observed by SEM. The polyphenol release profile was determined as a function of time. DPPH radical scavenging capacity was carried out for GPT/α-CD/aqCT gels to demonstrate their effective antioxidant. The non-cytotoxicity of GPT/α-CD/aqCT gels was confirmed by two-dimensional culture of human dermal fibroblast viability.

## 2. Results and Discussion

### 2.1. Preliminary Screening of Phytochemicals

Qualitative analysis of aqueous Cordyline terminalis Kunth leaf extract (aqCT) was performed to recognize the presence of alkaloids, glycosides (cardiac and coumarin glycosides), saponins, phenolic compounds, steroids, and phytosterols. As shown in Table 1, alkaloids, cardiac glycosides, saponins, and phenolic compounds were present in aqCT, while steroids and phytosterol were absent. These phytochemical families exhibited potential pharmacological effects [15]. Specifically, alkaloids possess anti-inflammatory, analgesic, antitumor, antioxidant, and antibacterial effects [16]. Coumarins have anticlotting, hypotensive, anti-inflammatory, antimicrobial, and antitumor activities [17]. Cardiac glycosides are commonly known as heart tonics, emetics, and diuretics in folk medicine, and anti-cancerous in recent reports [18]. Saponins exhibit their dual roles, a therapeutic ability due to their pharmacological effects, and an excipient because of inherently surfactant properties. Saponins can enhance the solubility of hydrophobic drugs and the skin’s permeability without affecting the lipid membrane [19]. Polyphenols are valuable to decrease the perils of the diseases for humans because of their unique properties, such as antibacterial, antiviral, antioxidant, and anti-inflammatory effects [20]. Interestingly, polyphenols can bind-block virus exterior protein receptors to inhibit viruses, as well as the severe acute respiratory syndrome coronavirus 2 (SARS-CoV-2) [20]. However, the applications of those bioactive compounds could meet some difficulties. First, the harmful effects might come from saponins (hemolysis) [17], coumarins (anti-coagulant effect) [19], and cardiac glycosides (toxicity) [18] if the high dosage is utilized. Second, the bioactive compounds are unstable in physiological conditions or vastly scattered after treatment [11]. To overcome two of these problems, the aqCT extract containing those bioactive compounds was encapsulated inside the gel matrices. As a result, the bioactive compounds were protected from degradation. They then sustainably released a small amount to prolong the treatment effects, while their low dosage could not cause the side effects. Moreover, the aqCT encapsulated gels could support the treatment to focus only at the wound site that also leads to minimizing the systemic influences.

### 2.2. Hydrogel Formation and Characterization

**Figure 1:**
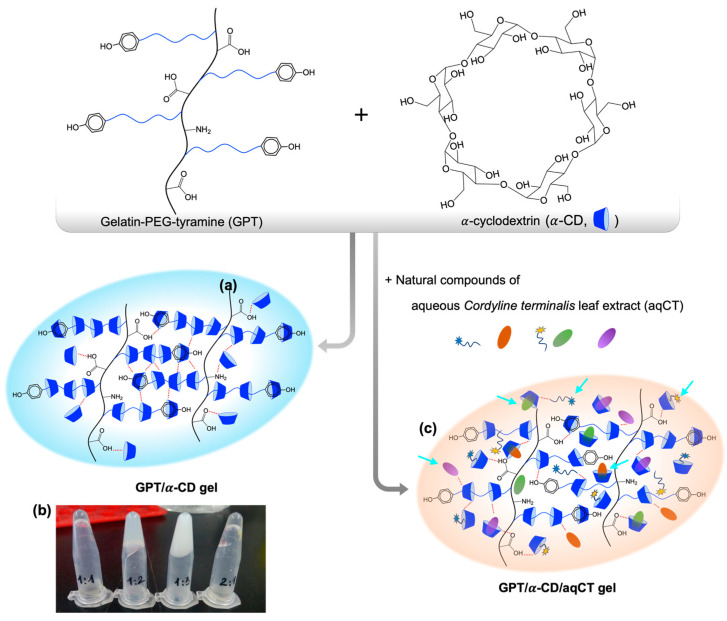
Schematic illustration of the gel formation: gelatin–polyethylene glycol (PEG)–tyramine (GPT) and α-cyclodextrin (α-CD) were mixed together to fabricate GPT/α-CD gel (**a**). Different GPT/α-CD ratios of 1:1, 1:2, 1:3, and 2:1 were tested, and only two formulas of 1:2 and 1:3 were gelled (**b**). The GPT/α-CD/aqCT gel was fabricated from GPT/α-CD gel encapsulating aqueous *Cordyline terminalis* leaf extract (aqCT) containing various natural compounds, including alkaloids, cardiac glycosides, saponins, and phenolic compounds (**c**).

Gelatin–polyethylene glycol–tyramine polymers (GPT) were synthesized by a two-step reaction; then its structure was confirmed by ^1^H NMR spectra and ultraviolet-visible spectrophotometry as previously reported [8,21]. The tyramine content of GPT was directly proportional to the PEG substitute degree. GPT and GPT’ contained 151.5 ± 1.3 μmol/g and 99.8 ± 3.7 μmol/g of tyramine moieties, which implied that the PEG substitution of GPT was drastically higher than GPT’. Hydrogels were formed by simply mixing GPT and α-CD solutions (Figure 1a). The driving forces that induced the formation of GPT/α-CD hydrogels were physical interactions, including hydrogen bonding and host-guest complex. Specifically, α-CD cavity could include the aromatic part [22]; thus, α-CDs were trapped on the tyramine head of the PEG chain attached to gelatin backbones. Abundant hydroxyl groups of α-CDs provided the source for hydrogen bonding with carboxylic groups on gelatin backbone and other hydroxyls of adjacent α-CDs. Additionally, α-CDs could thread along the PEG axle through an inclusion complex [23]. These threaded α-CDs on PEG branches of GPT were crosslinked through hydrogen bonding.

To explore the effect of the GPT:α-CD ratio on hydrogel formation, four mixtures with different ratios (1:1; 1:2; 1:3; and 2:1) were prepared. As shown in Figure 1b and Table 2, the GPT:α-CD ratios of 1:2 and 1:3 could be gelled after 135.6 ± 8.1 and 53.8 ± 5.2 min, respectively. These results showed that the GPT/α-CD gels could solidify rapidly when increasing α-CD amount. This is because α-CD played a role of crosslinker, supporting both host–guest inclusion and hydrogen bonding to crosslink the GPT precursors to form the gels. Therefore, increasing α-CD concentration could accelerate the gel formation due to high density of host–guest complexation and hydrogen bonding. However, their gelation time was not finely tuned. Indeed, the mixtures with different GPT:α-CD ratios of 1:2.4, 1:2.6, 1:2.8, and 1:4 were gelled after 100.6 ± 9.1, 60.4 ± 6.3, 55.5 ± 6.1, and 40.7 ± 10.2 min (Table 2). This characteristic was considered as one of the disadvantages of physically formed hydrogels.

To explore the effect of PEG content on gel formation, GPT’ containing a low PEG substitution was used to fabricate the GPT’:α-CD gels with the GPT’:α-CD ratios of 1:2 and 1:3. However, these two gels were not formed, while the GPT with high PEG content could be gelled at those same ratios. Therefore, the PEG substitution indirectly recognized by tyramine content immensely affected gel formation. Next, we fixed the solid concentration and tyramine/PEG content but replaced a part of α-CD with β-CD. Specifically, the final mixture named GPT3.75α-CD9.45β-CD1.05 was composed of 3.75 wt% of GPT, 9.45 wt% of α-CD, and 1.05 wt% of β-CD. The gelation time of GPT3.75α-CD9.45β-CD1.05 gel was 78.8 ± 13.5 min, which was 1.4-fold longer than GPT3.75α-CD10.5 gels. This observation revealed that α-CD played an essential role in GPT/CD gel formation. It could be explained by the host–guest formation between PEG chains and α-CD being more stable than β-CD [22]. Besides, β-CD possesses a suitable structure for intramolecular hydrogen bonding limiting the crosslinks for adjacent precursors. Altogether, GPT/CD gel formation was governed drastically by PEG substitution and cyclodextrin types.

The GPT/α-CD hydrogels with the ratio of 1:2.8 (coded as GPT3.75α-CD10.5) were utilized to incorporate the natural compounds of aqueous *Cordyline terminalis* Kunth leaf extract (Figure 1c). GPT and aqCT solutions were mixed homogeneously before adding α-CD, and then the mixtures were incubated at room temperature until gelling. GPT3.75α-CD10.5aqCT3.1, GPT3.75α-CD10.5aqCT6.2, and GPT3.75α-CD10.5aqCT12.5 hydrogels were gelled after 85.1 ± 9.4, 101.9 ± 11.2, and 297.7 ± 4.3 min, respectively (Table 2). These results implied that the presence of aqueous *Cordyline terminalis* Kunth leaf extract delayed the gelation time significantly. Increasing aqCT concentration from 3.1 to 12.5 mg/mL could prolong the gelation time of GPT3.75α-CD10.5 hydrogels from 1.6 to 5.4-fold. This phenomenon could be explained by the competition of bioactive compounds to hydrogel networks. The natural compounds that possessed the aromatic parts could be trapped inside α-CD cavities, which disturbed the formation of α-CD-threaded PEG axle. Other bioactive substances with polar groups, such as O-H, could form hydrogen bonding with that of α-CD. Besides, there were also some substances without any linkages, which were trapped due to gel matrices. Thus, they hurdled the hydrogen bonding interaction between GPT and α-CD. As a result, GPT/α-CD/aqCT hydrogel was postponed more slowly than GPT/α-CD hydrogels.

In the case of encapsulating high content of aqueous *Cordyline terminalis* Kunth leaf extract (aqCT), the GPT3.75α-CD15 hydrogels with an increased α-CD amount were applied. The gelation time of GPT3.75α-CD15aqCT9.4, GPT3.75α-CD15aqCT12.5, and GPT3.75α-CD15aqCT15.6 hydrogels were 51.1 ± 5.1, 59.6 ± 8.7, and 190.5 ± 5.3 min, respectively (Table 2). The presence of 9.4, 12.5, and 15.6 mg/mL aqCT extract delayed the gelation time of GPT3.75α-CD15 hydrogels about 1.3-, 1,5-, and 4.7-fold. Altogether, the effect of aqCT extract on the gelation time of GPT3.75α-CD15 hydrogels was less than GPT3.73α-CD10.5 ones. These results implied that the use of a high α-CD amount could mitigate the prolonged gelation of GPT/α-CD hydrogels in the presence of aqCT extract. The additional α-CD content might compensate for its interaction with natural compounds, especially host–guest inclusion. The high concentration of aqCT extract could be encapsulated, and the hydrogels should fabricate with high α-CD content.

**Figure 2:**
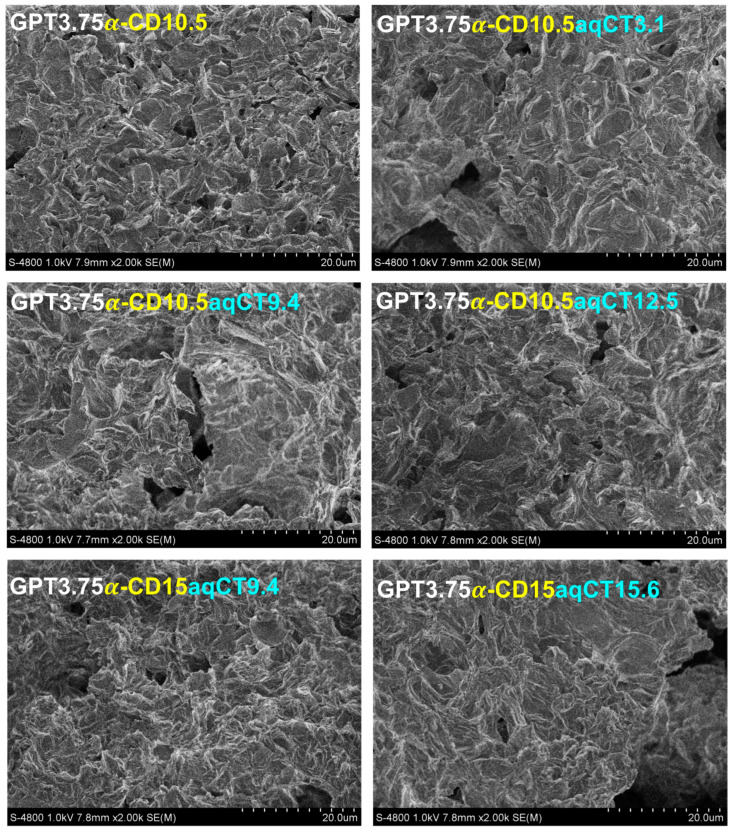
SEM micrographs of the cross-sectional gels, including GPT3.75α-CD10.5, GPT3.75α-CD10.5aqCT3.1, GPT3.75α-CD10.5aqCT9.4, GPT3.75α-CD10.5aqCT12.5, GPT3.75α-CD15aqCT9.4, and GPT3.75α-CD15aqCT15.6.

To explore the inner structure, six gel samples, including GPT3.75α-CD10.5, GPT3.75α-CD10.5aqCT3.1, GPT3.75α-CD10.5aqCT9.4, GPT3.75α-CD10.5aqCT12.5, GPT3.75α-CD15aqCT9.4, and GPT3.75α-CD15aqCT15.6 gels, were observed their cross-section by SEM. Figure 2 showed the micro-morphology of all tested gels. Their gel matrices exhibited an array of corrugation, cavities, and sockets that indicates the porosity of materials. The porous holes of GPT3.75α-CD10.5 gel were more regular than other gels containing aqCT extract. All GPT3.75α-CD10.5aqCT3.1, GPT3.75α-CD10.5aqCT9.4, GPT3.75α-CD10.5aqCT12.5, GPT3.75α-CD15aqCT9.4, and GPT3.75α-CD15aqCT15.6 gels showed a few significantly big cavities. It implied that the network of GPT/α-CD/aqCT gels was less homogeneous than GPT/α-CD ones without aqCT extract. This observation reinforced the hypothesis related to the obstruction of aqCT to GPT/α-CD/aqCT gel networks caused by complexation competition with α-CD. Therefore, this result and explanation corresponded with the gelation time data above. Moreover, it was realized that the properties of physical gels were sensitive to encapsulated agents. However, the microarchitecture and high porosity of all GPT/α-CD/aqCT gels were achieved. This is the favorable characteristic for biomedical applications. Water, nutrients, oxygen, and wastes could be exchanged throughout gel matrices. The drugs and cells could be encapsulated in gels without much stress due to tight networks. The drug release mechanism by diffusion was carried out more effectively.

### 2.3. Polyphenol Release

**Figure 3:**
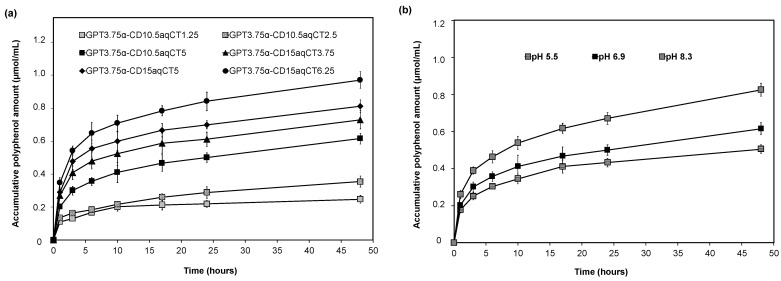
The cumulative polyphenol release profiles: released polyphenols from various GPT/α-CD/aqCT gels in DIW (pH 6.9) (**a**), released polyphenols from GPT3.75α-CD10.5aqCT5 gel under different pH (**b**).

The aqueous *Cordyline terminalis* Kunth leaf extract (aqCT) contained 1587.13 ± 94.32 μmol/g of polyphenols, which was determined by the Folin–Ciocalteu assay. Besides, the different solvents, including hexane, ethyl acetate, and ethanol, were also used to extract the *Cordyline terminalis* Kunth leaves. However, the polyphenol contents in those extracts were 227.78 ± 9.42, 500.07 ± 31.47, 930.49 ± 78.50 μmol/g, which were significantly less than that in aqCT. Thus, aqCT was selected to encapsulate inside GPT/α-CD gels to create the polyphenol release scaffold for biomedical applications. Six formulations with various aqCT amounts and GPT/α-CD ratios were prepared to record the polyphenol releasing profile (Figure 3). Three gels, including GPT3.75α-CD10.5aqCT1.25, GPT3.75α-CD10.5aqCT2.5, and GPT3.75α-CD10.5aqCT5, with the same GPT/α-CD ratios and an increased aqCT amount, could release polyphenols of 0.11, 0.14, and 0.20 μmol/mL for the first hour, respectively. Three other gels, such as GPT3.75α-CD15aqCT3.75, GPT3.75α-CD15aqCT5, and GPT3.75α-CD15aqCT6.25 also released 0.27, 0.29, and 0.35 μmol/mL, respectively. And then, for the next two hours, 0.019, 0.029, 0.098, 0.138, 0.186, and 0.195 μmol/mL of polyphenols were leaked from the GPT3.75α-CD10.5aqCT1.25, GPT3.75α-CD10.5aqCT2.5, GPT3.75α-CD10.5aqCT5, GPT3.75α-CD15aqCT3.75, GPT3.75α-CD15aqCT5, and GPT3.75α-CD15aqCT6.25 gels, respectively. More later, the released amount from these gels was decreased. For example, at the tenth hour, only 0.0055, 0.0084, 0.0140, 0.0110, 0.0112, and 0.0202 μmol/mL of polyphenols were released from the above gels. The released polyphenols were down to 0.0011–0.0091 μmol/mL/hour from the twenty-fourth hour. The release rate of polyphenols was decreased when the incubation time was long, which was explained by the slow diffusion of polyphenols incorporated deeply inside gel matrices. Then, the polyphenol was still released gradually from those gel matrices. Compared to the initial polyphenol amount loaded inside each gel formulation, the total polyphenol was released out of those gel matrices by about 8–13%. These results confirmed that the polyphenols could be released from GPT/α-CD/aqCT gels within the prolonged period. Besides, the gel matrices also governed the released polyphenol profile. Making a comparison between GPT3.75α-CD10.5aqCT5 and GPT3.75α-CD15aqCT5 gels encapsulating the same initial aqCT extract, it was realized that the gels made from the high amount of α-CD could release a high amount of polyphenols. Considering the gel network, high α-CD content could form the tight crosslinking. So, small molecules, such as polyphenols, were readily expelled from the gel matrices. Altogether, the sustained release of polyphenols could be controlled by the initial aqCT content and gel properties. Thus, it is simple to achieve the low demand of polyphenols (1 μM) for cell signaling pathways and metabolic processes [13].

The polyphenol release profile of GPT3.75α-CD10.5aqCT5 gels was carried out at different pH levels to understand the effect of pH on polyphenol release (Figure 3b). For the first hour, the released polyphenols were 0.18, 0.20, and 0.26 μmol/mL at pH 5.5, 6.9, and 8.3, respectively. Then the polyphenols were continuously released from GPT3.75α-CD10.5aqCT5 gels at the following hours. For example, the released polyphenols were 0.011, 0.014, and 0.019 μmol/mL/h at the tenth hour; and then 0.006, 0.010, 0.016 μmol/mL/hour at the forty-eighth hour, at pH 5.5, 6.9, and 8.3, respectively. It is realized that the released polyphenols were increased in the media with a high pH value. The alkaline environments caused a higher release rate of polyphenols from the gel matrices than the acidic ones. This phenomenon could be explained by the properties of supramolecular GPT/α-CD gels governed by pH values. The gelatin backbones of GPT polymers remained as almost carboxylic groups because amine groups were reacted to link with PEG–tyramine. Therefore, in the media having higher pH values, carboxylic groups of GPT were ionized, which led to the appearance of charge repulsion in the gel networks. Thus, the conformation of gels was expanded, resulting in an easy escape of polyphenols. On the contrary, at lower pH values, the carboxylic groups were not deprotonated, and they could interact together and with hydroxyl groups of α-CD through hydrogen bonding. As a result, the GPT/α-CD gels were reinforced and became a compact conformation in the acidic conditions. Thus, the polyphenols inside gels would be hindered and release slowly.

### 2.4. Antioxidant Test

**Figure 4:**
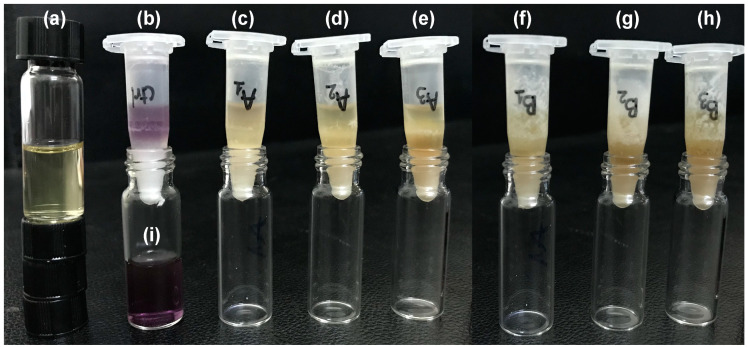
The color change of DPPH scavenging tests after incubation with Vitamin C solution of 1 mg/mL (**a**), GPT3.75α-CD10.5 gel—a control sample (**b**), GPT3.75α-CD10.5aqCT1.25 gel (**c**), GPT3.75α-CD10.5aqCT2.5 gel (**d**), GPT3.75α-CD10.5aqCT5 gel (**e**), GPT3.75α-CD15aqCT3.75 gel (**f**), GPT3.75α-CD15aqCT5 gel (**g**), GPT3.75α-CD15aqCT6.25 gel (**h**), and DIW (**i**).

DPPH radical scavenging activity of GPT/α-CD/aqCT gels was determined to evaluate their antioxidant activity. The DPPH˙ radical is a long-lived organic nitrogen radical with a purple color. It is mixed with an antioxidant/reducing compound; the reaction will occur and turns DPPH˙ radical into corresponding hydrazine with a yellow color. DPPH˙ radical solutions showed purple color and were turned yellow when reacting completely with antioxidant agents, such as vitamin C (Figure 4). In the case of an incomplete reaction, the purple intensity would be decreased; absorbance at 517 nm was measured to quantify the DPPH radical scavenging level. Herein, seven gel formulations were prepared and incubated in DPPH radical solutions. GPT3.75α-CD10.5 gels without aqCT extract showed purple (Figure 4b) but lighter than the primary color of DPPH radical solution (Figure 4i). The scavenging percentage of GPT3.75α-CD10.5 gels was 16%. Their scavenging capacity against DPPH radicals was attributed to phenol moieties and the hydrogen-donating ability of cysteine on gelatin backbones [24]. GPT3.75α-CD10.5aqCT1.25, GPT3.75α-CD10.5aqCT2.5, GPT3.75α-CD10.5aqCT5, GPT3.75α-CD15aqCT3.75, GPT3.75α-CD15aqCT5, and GPT3.75α-CD15aqCT6.25 gels containing various amounts of aqCT extract from 1.25 to 6.25 mg/mL completely decolorized the DPPH radical solutions (Figure 4c–h). The introduction of aqCT improved the DPPH scavenging capacity of GPT3.75α-CD10.5 gels tremendously to 100%. This highly antioxidant activity of aqCT is due to the phytochemicals, especially abundant polyphenols. Polyphenols are a large family of phytoconstituents and are composed of multiple phenol units. Polyphenols possess an antioxidant activity being better than or comparable to vitamin E [13]. Furthermore, polyphenols can effectively scavenge reactive oxygen species (ROS) because they are easily oxidized to produce the ortho-quinones. Recently, polyphenols have been demonstrated as a modulator of various enzymes in the regulating of cellular survival and the stress response [13].

It is evident that GPT/α-CD/aqCT gels exhibited excellent antioxidant activity, which property is beneficial for wound healing acceleration [25]. Reactive oxygen species (ROS) were increased in chronic wounds, such as diabetic foot ulcers, pressure ulcers, and venous/arterial leg ulcers [26]. Excessive ROS could cause damages to cells and impair the wound healing progress. Thus, the GPT/α-CD/aqCT gels could scavenge ROS to create balanced environments for effective wound healing. In this regard, topical application of GPT/α-CD/aqCT gels on acute wounds has been suggested as ROS-manipulating products to enhance the wound-healing response.

### 2.5. Cell Study

**Figure 5:**
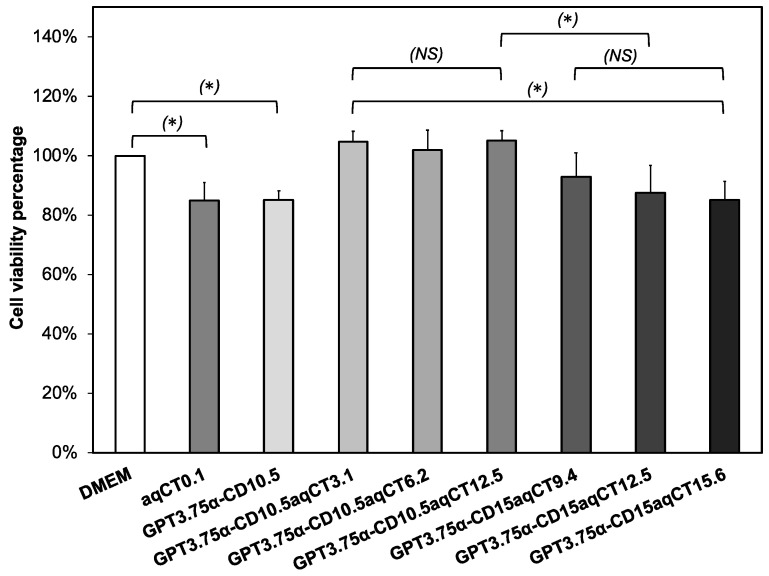
Viability of human dermal fibroblasts (hDFBs) was quantitatively analyzed by WST-1 assay. The hDFBs were incubated with DMEM media (DMEM), the aqCT solution of 0.1 mg/mL, the gel extracts of GPT3.75α-CD10.5, GPT3.75α-CD10.5aqCT3.1, GPT3.75α-CD10.5aqCT6.2, GPT3.75α-CD10.5aqCT12.5, GPT3.75α-CD15aqCT9.4, GPT3.75α-CD15aqCT12.5, and GPT3.75α-CD15aqCT15.6 for 24 h (* *p* < 0.05: statistical significance, *NS*: nonstatistical significance).

The cytotoxicity of GPT/α-CD/aqCT gels was accessed by incubating their extracts with human dermal fibroblast cells (hDFBs) for 24 h. The control samples were set up for hDFBs contacting pure DMEM and aqCT solutions of 0.1 mg/mL. The WST-1 assay was performed to determine the cell viability percentage in those conditions. As shown in Figure 5, aqCT solutions (0.1 mg/mL) and all gel samples achieved a cell viability percentage of more than 80%, indicating an acceptable level of cytocompatibility. Interestingly, GPT3.75α-CD10.5aqCT3.1, GPT3.75α-CD10.5aqCT6.2, and GPT3.75α-CD10.5aqCT12.5 gel extracts increased the hDFBs proliferation significantly, and their cell viability percentage gained 105 ± 4%, 102 ± 7%, and 105 ± 3%, respectively. These values were higher than that of GPT3.75α-CD10.5 gels without aqCT incorporation about 18–20%. The introduction of aqCT was beneficial for cell proliferation to GPT3.75α-CD10 gels. In addition, these GPT3.75α-CD10.5 gels encapsulating 3.1–12.5 mg/mL aqCT extract showed the cell viability to be much higher than the pure aqCT at 0.1 mg/mL (85 ± 5%). It was recognized that gels could improve the biological activity of aqCT by controlling the release of small amounts of bioactive substances. However, GPT3.75α-CD15aqCT15.6 gels with a highly increased aqCT caused a decrease of cell viability down to 85 ± 6%. Besides, a huge increase of α-CD content also mitigates cell viability. Indeed, GPT3.75α-CD10.5aqCT12.5 got viability percentage being lower than that of GPT3.75α-CD10.5aqCT12.5 (87% vs. 105%). Altogether, aqCT showed a positive effect on cell proliferation when its concentrations were less than or equal to 12.5 mg/mL and had to be encapsulated in gels with a suitable α-CD amount.

## 3. Materials and Methods

### 3.1. Materials

Gelatin (from porcine skin, type A, >300 bloom, Sigma-Aldrich, Darmstadt, Germany), 1-ethyl-3-(3-dimethylamino propyl)-carbodiimide (EDC), 3-(4-hydroxyphenyl)propionic acid (HPA), N-hydroxysuccinimide (NHS), poly(ethylene glycol) (PEG, 4000 Da), 4-dimethylamino pyridine (DMAP), alpha-cyclodextrin (α-CD), beta-cyclodextrin (β-CD), p-nitrophenyl chloroformate (PNC), and Folin and Ciocalteu’s reagent were bought from Sigma–Aldrich (St. Louis, MO, USA). Tyramine (TA) was supplied by ACROS Organic (A Thermo Fisher Scientific Brand, Waltham, MA, USA). Triethylamine (TEA) was purchased by Kanto Chemical Co. (Osaka, Japan).

Potassium iodide (KI, ACS reagent, 99.0%), potassium hydroxide (KOH, reagent grade, 90%), iodine (I_2_, ACS reagent, 99%), acetic anhydride (Ac_2_O, 99%), iron chloride reagent grade (FeCl_3_, 97%), sodium carbonate (Na_2_CO_3_, ACS reagent, 99.5%), and 2,2-di(4-tert-octylphenyl)-1-picrylhydrazyl (free radical, DPPH) were purchased from Merck KGaA (Darmstadt, Germany). Hydrochloride acid (HCl, 36–38%), glacial acetic acid (CH_3_COOH, 98%), concentrated sulfuric acid (H_2_SO_4_, 98%), and ethanol (EtOH, 95%) were obtained from Xilong Scientific Co., Ltd. (Shantou, China). Cordyline terminalis Kunth leaves were harvested from Dak Lak province, Vietnam.

Human dermal fibroblasts (hDFBs) were purchased from Lonza Inc. (Walkersville, MD, USA). Dulbecco’s modified Eagle medium (DMEM), trypsin/ethylenediaminetetraacetic acid (EDTA), and penicillin−streptomycin (P/S) were obtained from Gibco BRL (Grand Island, New York, NY, USA). Dulbecco’s phosphate buffered saline (DPBS) and fetal bovine serum (FBS) were supplied by Wisent (Saint-Bruno, QC, Canada). WST-1 assay kit (cell proliferation) was obtained from Merck KGaA (Darmstadt, Germany).

### 3.2. Aqueous Extract Preparation and Preliminary Screening of Phytochemicals

#### 3.2.1. Aqueous Extract Preparation

The collected Cordyline terminalis Kunth leaves without diseases were washed carefully with distilled water (DIW) and sundried until humidity less than 5 weight percentage (wt%). Dried leaves were ground into fine powder. The leaf powder was immersed in DIW at a weight/volume ratio of 1/1. After boiling for 3 h, the mixture was cooled and filtrated with a Buchner funnel and vacuum pump. The filtrated solution was freeze-dried to obtain the aqueous crude extract of Cordyline terminalis Kunth leaves, which was named aqCT.

#### 3.2.2. Preliminary Screening of Phytochemicals

The aqCT solution was prepared at 1 wt% in DIW for phytochemical screening.

Alkaloid identification: Wagner’s reagent was prepared by dissolving 2 g of I_2_ and 6 g of KI in 100 mL of DIW [27]. The aqCT solution 1 wt% was mixed with dilute hydrochloride acid and Wagner’s reagent at a volume ratio of 1:1:1. The appearance of brownish precipitation indicates the presence of alkaloids.

Cardiac glycoside identification (Keller–Kiliani test): The aqCT solution 1 wt% was mixed with glacial acetic acid, FeCl_3_ solution 5 wt%, and concentrated sulfuric acid at a volume ratio of 1:1:1:1. If the interface of two layers appeared a reddish-brown color, there was the presence of cardiac glycosides [28].

Saponin identification: A foam test was performed to determine the presence of saponins [29]. The aqCT solution 1 wt% was diluted 4-fold with DIW. The diluted aqCT solution was shaken in a test tube. The froth was formed and stable for at least 5 min that indicates the presence of saponins.

Coumarin glycoside identification: The aqCT solution 1 wt% (1 mL) was mixed with 1 mL of ethanol. This mixture was adjusted to basic pH by a few drops of potassium hydroxide solution (0.5 N in ethanol). Coumarin glycosides were present if the blue/violet/brown/green or yellow fluorescence appeared.

Phenolic compound identification: The aqCT solution 1 wt% (1 mL) was reacted with FeCl_3_ solution (5 wt% in ethanol) [29]. The reacted mixture was changed to a bluish dark color that indicates the presence of phenolic compounds.

Steroid identification: The aqCT solution 1 wt% (1 mL) was mixed with chloroform (1 mL), anhydric acetic (1 mL), and two drops of concentrated sulfuric acid. If there were steroids in aqCT, the mixture would turn to red, blue, and green color respectively.

Phytosterol identification: Few drops of glacial acetic acid were added into the aqCT solution 1 wt% (1 mL). Then anhydride acetic (3 mL) and a few drops of concentrated sulfuric acid were added to the mixture. If the bluish-green color appeared, the presence of phytosterols was confirmed.

### 3.3. Polymer Synthesis

Gelatin–polyethylene glycol–tyramine (GPT) was synthesized according to a previous study [8]. In brief, DMAP (0.916 g) and TEA (0.759 g) were dissolved in 20 mL of methylene chloride. This homogeneous solution was poured into a PEG solution (10 g of PEG in 100 mL of methylene chloride). The reaction was performed for 15 min at room temperature (RT). After, this reacted mixture was dropped into a PNC solution (1.512 g of PNC in 20 mL of methylene chloride). This reaction was carried out for 24 h under nitrogen media to obtain a PNC–PEG–PNC product purified by precipitating in cold diethyl ether and drying under vacuum. Then, PNC–PEG–PNC solution (0.83 mmol in dimethyl sulfoxide) was reacted with TA solution (0.52 mmol in dimethyl sulfoxide) at RT under nitrogen atmosphere to obtain TA–PEG–PNC. After 6 h, this reacted mixture was poured into gelatin solution (1 g of gelatin in 300 mL of dimethyl sulfoxide) to graft TA–PEG–PNC onto gelatin backbone at 40 °C under nitrogen atmosphere. After 16 h, the entire mixture was transferred into a dialysis bag (molecular cut-off = 6000–8000 kDa). The dialysis procedure was carried out for 3 days. The dialysis media was changed continuously at least 3 times per day. Finally, the dialyzed product was freeze-dried to obtain the GPT product. Tyramine amount was determined quantitatively by ultraviolet-visible spectrophotometry and standard curve method. The degree of substitution of tyramine moieties of GPT was 151.5 ± 1.3 μmol/g. Another protocol of GPT synthesis was carried out similarly but used the low amount of PNC–PEG–PNC (0.2 mmol) and tyramine (0.2 mmol). This obtained product was named GPT’. The degree of substitution of tyramine moieties of GPT’ was 99.8 ± 3.7 μmol/g.

### 3.4. Hydrogel Formation and Characterization

GPT solutions of 20 wt%, α-CD solutions of 14 wt% and 20 wt% were prepared in DIW. AqCT solutions were prepared in DIW at various concentrations, including 0.05 g/mL, 0.10 g/mL, 0.15 g/mL, 0.20 g/mL, and 0.25 g/mL. In the microtube, GPT of 20 wt% (75 μL), DIW (25 μL), and α-CD solutions of 14 wt% (300 μL) were mixed and sonicated for 15 min to fabricate the GPT3.75α-CD10.5 hydrogels. This mixture was kept at RT until no flow was observed. In the case of GPT/α-CD hydrogels encapsulating aqCT extract, the fabrication procedure was carried out as GPT3.75α-CD10.5 hydrogels, but various aqCT extract solutions replaced DIW (25 μL). For example, to form GPT3.75α-CD10.5aqCT3.1 hydrogels, GPT of 20 wt% (75 μL), aqCT solution of 0.05 g/mL (25 μL) and α-CD solutions of 14 wt% (300 μL) were mixed and sonicated for 15 min. The numerical part in hydrogel code indicated the final concentration of GPT and α-CD in wt%, and the final aqCT concentration in mg/mL. Similarly, other hydrogels were formed; the specific formulations and corresponding code are presented in Table 3. The time when the mixtures stopped flowing was recorded as the gelation time [30,31]. Each sample was triplicated to calculate the average value and standard deviation.

For the scanning electron microscopy (SEM), all hydrogels in Table 1 were fabricated in cubic molds. After freeze-drying, the hydrogel cubes were crosslinked. SEM (S-4800, Hitachi, Okinawa, Japan) was operated to observe the cross-section of those samples. An applied voltage was 1 kV.

### 3.5. Polyphenol Release

The release of polyphenols was carried out by direct contact between the gels and the media [32,33]. Six types of hydrogels, including GPT3.75α-CD10.5aqCT1.25, GPT3.75α-CD10.5aqCT2.5, GPT3.75α-CD10.5aqCT5, GPT3.75α-CD15aqCT3.75, GPT3.75α-CD15aqCT5, and GPT3.75α-CD15aqCT6.25, were formed in the Eppendorf tube with the total volume of 400 μL. Each type was repeated three times. After 3 h for hydrogel stabilization, 1 mL of DIW (pH 6.9) was added into each sample to extract the polyphenol released amount. Then, after each specific time interval of 1, 3, 6, 10, 17, 24, and 48 h, the medium (1 mL) was sampled for polyphenolic determination, and 1 mL of DIW was added for the next sampling.

Similarly, the polyphenol release experiment of GPT3.75α-CD10.5aqCT5 gel samples was performed in different pH media. The pH 5.5 buffer was prepared from 272 g of sodium acetate and 6 mL of glacial acetic acid in 1000 mL of DIW. The pH 8.3 solution was prepared from 6.18 g of boric acid, 9.54 g of sodium tetraborate, and 4.38 g of sodium chloride in 1000 mL of DIW.

Folin and Ciocalteu’s method was utilized to measure the polyphenolic content [34]. Gallic acid was used to prepare the standard solutions at various concentrations (0.1, 0.5, 1.0, 5.0, 10.0 µg/mL). Folin and Ciocalteu’s reagent 0.2 M (1 mL) was reacted with 0.5 mL of samples or gallic acid standard solutions in test tubes. All these mixtures were vortexed and incubated for 2 min. After adding Na_2_CO_3_ solution 700 mM (1.5 mL), the reaction was incubated at RT for 2 h. The optical density of reacted solutions was read at 765 nm using a UV–Vis spectrophotometer (Shimadzu UV-1800, Kyoto, Japan). The polyphenolic content in samples was calculated by the gallic acid standard curve.

### 3.6. Antioxidant Test

DPPH solution was prepared in ethanol at 0.5 mM [24], stored in dark conditions at 4 °C. Hydrogel samples were formed in the Eppendorf tube. Total hydrogel volume was 400 µL. After 3 h for stabilization, 1 mL of DPPH solution was added to each hydrogel sample. Vitamin C solution was used as a positive control, 1 mL of Vitamin C (1 mg/mL) was mixed with 1 mL of DPPH solution. The control indicated no radical scavenging for the DPPH solution, including 1 mL of DPPH 0.004 wt% diluted with 400 µL of DIW. After incubation for 30 min in dark conditions, the reacted DPPH solutions read an optical density at 517 nm using UV–Vis spectrophotometer (Shimadzu UV-1800, USA). The solution including 400 µL of DIW and 1 mL of ethanol was blank. The optical density of DPPH treating hydrogels was symbolized as OD_sample_, and OD_DPPH_ was of the DPPH solution and DIW. The scavenging percentage of each sample was calculated as the following formula:

$$\mathrm{Scavenging}\;\mathrm{percentage}=\frac{{\mathrm{OD}}_{\mathrm{DPPH}}-{\mathrm{OD}}_{\mathrm{sample}}}{{\mathrm{OD}}_{\mathrm{DPPH}}}\times 100\%$$

### 3.7. Cell Study

Human dermal fibroblasts (hDFBs) were seeded on a 48-well plate at a density of 10^4^ cells/well. After 3 days of incubation at 37 °C under 5% CO_2_ atmosphere, all hDFBs attached and fully spread on the bottom surface of the well plate. To prepare the hydrogel samples for cell study, 400 μL of each hydrogel including GPT3.75α-CD10.5, GPT3.75α-CD10.5aqCT3.1, GPT3.75α-CD10.5aqCT6.2, GPT3.75α-CD10.5aqCT12.5, GPT3.75α-CD15aqCT9.4, GPT3.75α-CD15aqCT12.5, and GPT3.75α-CD15aqCT15.6 was respectively fabricated in Eppendorf tubes. DMEM (1 mL) was added to each hydrogel and incubated for 24 h. These hydrogel extracts were utilized to treat the hDFBs proliferated in a 48-well plate. The hDFBs were cultured with pure DMEM that was a control sample. The aqCT solution of 0.1 mg/mL was utilized to test the cytotoxicity of crude extract. After 24 h, all hDFBs were washed with DPBS carefully. WST-1 reagent and DMEM (1:1 volume ratio) were added into each hDFBs well and incubated for 2 h at 37 °C under 5% CO_2_ atmosphere. After, the WST-1-reacted media (100 μL) was transferred into the 96-well plate. Optical density (OD) was recorded at 450 nm by a microplate reader (Cytation^TM^ 3 Cell Imaging Multi-Mode Reader, BioTek^TM^, Winooski, VT, USA). A blank solution was prepared from DMEM and WST-1 reagent (1/1 volume ratio) without contacting cells. The viability percentage was calculated as the following formula:

$$\mathrm{Viability}\;\mathrm{percentage}=\frac{{\mathrm{OD}}_{\mathrm{sample}}-{\mathrm{OD}}_{\mathrm{blank}}}{{\mathrm{OD}}_{\mathrm{control}}-{\mathrm{OD}}_{\mathrm{blank}}}\times 100\%$$

### 3.8. Statistical Analysis

All experiments were carried out in triplicate. The obtained results were expressed as the mean ± standard deviation (SD). Statistical analyses were performed by the Student’s *t*-test [35]. Statistical differences were considered to be significant when *p*-value was less than 0.05. Vice versa, *p*-value was more extensive than 0.05, and the difference between the two values was nonstatistical significant (NS).

## 4. Conclusions

In summary, we presented an interesting supramolecular gel fabricated from gelatin–polyethylene glycol–tyramine (GPT) and α-cyclodextrin (α-CD) for encapsulating an aqueous *Cordyline terminalis* Kunth leaf extract (aqCT). It was determined that GPT with a phenol content of 151.5 μmol/g and GPT/α-CD ratios of less than or equal to 0.5 could form the gel with α-CD through inclusion complex and hydrogen bonding. The gelation time was around one hour or longer depending on a decrease of α-CD amount. The aqCT solutions containing bioactive compounds (alkaloids, cardiac glycosides, coumarin glycosides, saponins, and phenolic compounds) disturbed the physical gel networks due to interacting competition. Thus, GPT/α-CD/aqCT gels were formed more slowly than GPT/α-CD ones. By SEM observation, GPT/α-CD and GPT/α-CD/aqCT gels possessed microarchitecture and high porosity, but GPT/α-CD/aqCT had more irregular cavities than another. GPT/α-CD/aqCT gels could sustainably release natural polyphenols from a few nmol/mL to several μmol/mL depending on initial aqCT encapsulation and the used GPT/α-CD ratios. All GPT/α-CD/aqCT formulations have demonstrated to have high antioxidant activity through 100% scavenging DPPH radicals. Besides, those gels with or without aqCT extract were non-cytotoxicity. Their cell viability gained more than 85% versus DMEM media. Significantly, the GPT3.75α-CD10.5aqCT gels with aqCT amount of 3.1–12.5 mg/mL enhanced the cell proliferation more so than GPT3.75α-CD10.5 gel without extract. These findings would contribute to developing a biocompatible scaffold for drug delivery systems or other biomedical applications. However, these GPT/α-CD/aqCT systems were still gelled slowly, which should be improved further. Along with this, the GPT/α-CD/aqCT gels might be studied to integrate multi-properties, such as tunable mechanical strength, self-healing, adhesive, stimuli-responsive and antibacterial characteristics satisfying the requirements for advanced biomaterials.

## Figures and Tables

**Table 1:** Screening phytoconstituents in aqueous *Cordyline terminalis* Kunth leaf extract.

Phytochemicals	Method	Observation	Result
Alkaloids	Wagner test	Brown precipitation	+
Cardiac glycosides	Keller-Kiliani Test	Red interface between two layers	+
Coumarin glycosides	Alkaline test	Brown fluorescence	+
Saponins	Foam test	A stable froth	+
Phenolic compounds	FeCl_3_ test	Green dark color	+
Steroids	Liebermann-Burchard test	None	−
Phytosterol	Glacial acetic acid	None	−

“+”: positive result; “−“: negative result.

**Table 2:** Gelation time of various hydrogel formulations.

Hydrogels	Gelation Time (min)	Hydrogels	Gelation Time (min)
GPT3.75α-CD3.75 (GPT:α-CD = 1:1)	No gel	GPT’3.75α-CD7.5 (GPT’:α-CD = 1:2)	No gel
GPT3.75α-CD1.875 (GPT:α-CD = 2:1)	No gel	GPT’3.75α-CD11.25 (GPT’:α-CD = 1:3)	No gel
GPT3.75α-CD7.5 (GPT:α-CD = 1:2)	135.6 ± 8.1	GPT3.75α-CD10.5aqCT3.1	85.1 ± 9.4
GPT3.75α-CD9 (GPT:α-CD = 1:2.4)	100.6 ± 9.1	GPT3.75α-CD10.5 aqCT6.2	101.9 ± 11.2
GPT3.75α-CD9.75 (GPT:α-CD = 1:2.6)	60.4 ± 6.3	GPT3.75α-CD10.5 aqCT12.5	297.7 ± 4.3
GPT3.75α-CD10.5 (GPT:α-CD = 1:2.8)	55.5 ± 6.1	GPT3.75α-CD15 aqCT9.4	51.1 ± 5.1
GPT3.75α-CD11.25 (GPT:α-CD = 1:3)	53.8 ± 5.2	GPT3.75α-CD15 aqCT12.5	59.6 ± 8.7
GPT3.75α-CD15 (GPT:α-CD = 1:4)	40.7 ± 10.2	GPT3.75α-CD15 aqCT15.6	190.5 ± 5.3
GPT3.75α-CD9.45β-CD1.05	78.8 ± 13.5	-	-

**Table 3:** Hydrogel compositions.

Hydrogel Code	GPT 20 wt% (μL)	α-CD 14 wt% (μL)	α-CD 20 wt% (μL)	DIW (μL)	AqCT (g/mL, (μL)
GPT3.75α-CD10.5	75	300	-	25	-
GPT3.75α-CD10.5aqCT3.1	75	300	-	-	0.05, 25
GPT3.75α-CD10.5 aqCT6.2	75	300	-	-	0.10, 25
GPT3.75α-CD10.5 aqCT12.5	75	300	-	-	0.25, 25
GPT3.75α-CD15 aqCT9.4	75	-	300	-	0.15, 25
GPT3.75α-CD15 aqCT12.5	75	-	300	-	0.20, 25
GPT3.75α-CD15 aqCT15.6	75	-	300	-	0.25, 25

## Data Availability

The data presented in this study are available on request from the corresponding author.
